# A Computational Approach Using Bioinformatics to Screening Drug Targets for* Leishmania infantum* Species

**DOI:** 10.1155/2018/6813467

**Published:** 2018-03-28

**Authors:** Miguel Angel Chávez-Fumagalli, Mônica Santos Schneider, Daniela Pagliara Lage, Grasiele de Sousa Vieira Tavares, Débora Vasconcelos Costa Mendonça, Thaís Teodoro de Oliveira Santos, Rodrigo Maia Pádua, Ricardo Andrez Machado-de-Ávila, João Paulo Viana Leite, Eduardo Antonio Ferraz Coelho

**Affiliations:** ^1^Programa de Pós-Graduação em Ciências da Saúde: Infectologia e Medicina Tropical, Faculdade de Medicina, Universidade Federal de Minas Gerais, Belo Horizonte, MG, Brazil; ^2^Departamento de Produtos Farmacêuticos, Faculdade de Farmácia, Universidade Federal de Minas Gerais, 31.270-901 Belo Horizonte, MG, Brazil; ^3^Programa de Pós-Graduação em Ciências da Saúde, Universidade do Extremo Sul Catarinense, Criciúma, SC, Brazil; ^4^Departamento de Bioquímica e Biologia Molecular, Universidade Federal de Viçosa, 36.570-000 Viçosa, MG, Brazil

## Abstract

**Background:**

The development of new therapeutic strategies to treat patients for leishmaniasis has become a priority. The antileishmanial activity of the strychnobiflavone flavonoid was recently demonstrated against* Leishmania amazonensis* and* Leishmania infantum* amastigotes and promastigotes. The biological effect of this molecule was identified due to its capacity to interfere in the parasite mitochondrial membrane; however, the underlying molecular mechanism remains unclear.

**Methods and Results:**

In this study, a computational approach using bioinformatics was performed to screen biological targets of strychnobiflavone in* L. infantum*. Computational programs, such as the target fishing approach and molecular docking assays, were used. Results showed that the putative pathway targeted by strychnobiflavone in* L. infantum* is the methylglyoxal degradation superpathway, and one hydrolase-like protein was predicted to be the molecular target of this flavonoid in the parasites.

**Conclusion:**

In this context, this study provides the basis for understanding the mechanism of action of strychnobiflavone in* L. infantum* and presents a strategy based on bioinformatics programs to screen targets of other molecules with biological action against distinct pathogens.

## 1. Introduction

Visceral leishmaniasis (VL) is a potentially fatal disease caused by the protozoan* Leishmania infantum* found throughout the Mediterranean, Southwest Asia, China, Central America, and South America [1]. The parasites are transmitted by the bite of infected phlebotomine sand flies and can parasitize mammalian cells in organs, such as the hosts' spleen, bone marrow, and liver [2, 3]. The clinical manifestations of the disease vary from an asymptomatic infection to fatal visceral disease [4–6]. The parenteral administration of pentavalent antimonials continues to be the first choice as VL treatment; however, the occurrence of side effects, such as myalgias, arthralgias, chemical pancreatitis, and cardiotoxicity, has also been identified in patients [7].

Amphotericin B is an antifungal drug presenting antileishmanial activity; however, its clinical use is limited by the high toxicity and/or high cost of lipid-based formulations [8–10]. As a consequence, the search for new treatment products for VL is considered as a priority [11]. A number of natural product-derived compounds have shown a significant role against different diseases [12, 13]. Over the past decade, about 340 natural compounds were identified as having promising antileishmanial activity [14].

In this context, greater attention has been given to plants evaluation, seeking to identify new antileishmanial products [15, 16]. Plants present secondary products resulting from their metabolism, with well-defined chemical structures, representing a basis for new pharmaceuticals [17]. In addition, the wide variety of modern techniques of purification has allowed for the identification of new compounds that can in turn become effective antileishmanial products [18].

Recently, an ethyl acetate extract derived from* Strychnos pseudoquina* stem bark proved to be effective against different* Leishmania* species. Two flavonoids, quercetin 3-O-methyl ether and strychnobiflavone, were identified as the main responsible agents for this antileishmanial activity [19]. These molecules presented low toxicity in murine macrophages and a null hemolytic activity in human red blood cells. In a new study, the mechanism of action of strychnobiflavone in* L. infantum* proved to be related to alterations induced by this molecule in the parasite's mitochondrial membrane potential [20].

Aiming to screen the molecular target of this flavonoid in* L. infantum* by means of distinct bioinformatics programs, the present study applied a computational approach based on target fishing and molecular docking assay. Moreover, investigations of drug-drug and drug-human protein interactions were developed to evaluate the interactive mechanisms of this molecule with mammalian proteins, which could eventually cause adverse effects in the patients during antileishmanial treatment.

## 2. Material and Methods

### 2.1. Target Fishing Approach

Target fishing screen was based on chemical similarity, as well as on the use of current knowledge of the bioactivity of small molecules [21]. These methodologies were based on the “chemical similarity principle,” in which similar molecules are likely to have equivalent properties [22]. For this, the chemical structure of strychnobiflavone was retrieved from the PubChem database [23] and uploaded to the TargetHunter [24], SwissTargetPrediction [25], Similarity Ensemble Approach (SEA) [26], and PASS Online [27] servers. Threshold values were selected by default parameters, and molecular targets were considered as possible “hits,” when the four algorithms presented a consensual result.

### 2.2. Literature Review

Since the main tools offered by the servers to evaluate target fishing are related to human proteins, a cross-reference with* L. infantum*-related proteins was performed. For this, “hits” were employed as keywords in a literature review performed on the PubMed server (https://www.ncbi.nlm.nih.gov/pubmed), as described in [28]. Next, the obtained data were manually extracted, information about* L. infantum* metabolic pathways was retrieved from the Kyoto Encyclopedia of Genes and Genomes (KEGG) database [29], and a manual comparison was performed. The complete sequence-based pathway analysis of the information was retrieved from MetaCyc [30].

### 2.3. Protein-Protein Interaction Search

The proteins belonging to the predicted metabolic pathway were chosen to analyze their interaction with other molecules. For this, the Retrieval of Interacting Genes (STRING) program was employed. This server contains known and unknown protein associations, based not only on the direct and physical association of proteins, but also on their genetic interactions and involvement in subsequent catalysis steps in the metabolic processes [31]. All obtained sequences were selected for further analysis, and their FASTA sequences were retrieved from the UniProt database (http://www.uniprot.org/), using their identification numbers.

### 2.4. Protein Sequence Comparison

The* L. infantum* protein sequences obtained by using the STRING server were subjected to BLAST assay [32], and the sequence's similarity search was performed by using murine and human databases. The “expect” value (*e*-value) was lower than 0.005, and a minimum hit score higher than 100.0 was used to exclude homologous sequences. The proteins that showed hits with the aforementioned cut-off values were considered to be “nonhomologous” proteins [33–35] and were used in the subsequent analyses, while remaining sequences were excluded.

### 2.5. Homology Modeling

The amino acid sequences of the selected proteins were uploaded in a FASTA format to the Iterative Threading Assembly Refinement (I-TASSER) server. Tertiary structures were predicted in PDB format, and results showed five top models for each entry, where ones with the highest confidence score (*c*-score) represented the best model [36].

### 2.6. Druggable Pocket Identification

The active sites in the evaluated tertiary structures of selected proteins were identified by using the DoGSiteScorer server [37], in which the druggability of a pocket can be automatically predicted through the analyses of its size, shape, and chemical features. Considering all descriptors, the DoGSiteScorer server provides a drug score value (0-1) for a selected pocket, where a higher score and a druggable pocket were estimated.

### 2.7. Molecular Docking Assay

The tertiary structures predicted by the I-TASSER server were used to perform a docking assay in the strychnobiflavone structure by using the SwissDock server [38]. Binding modes were scored using their FullFitness and clustered. Clusters were ranked according to the average FullFitness of their elements, and results of the SwissDock were viewed using the UCSF Chimera package [39].

### 2.8. Functional Annotation of Hypothetical Proteins

The experimental strategy was developed as described in [40]. Briefly, the functional domain of selected proteins was evaluated by the following programs: Pfam [41], PANTHER 10.0 [42], SUPERFAMILY [43], SMART [44], CATH [45], and ProtoNet 6.0 [46]. The Receiver Operator Characteristic (ROC) curves were constructed to estimate the protein localization and function in the parasite. Results were expressed as sensitivity (Se), specificity (Sp), accuracy (Ac), and area under the curve (AUC).

### 2.9. Chemical-Protein Interactome Profile of Strychnobiflavone

The chemical-protein interactome (CPI) refers to the information of interaction of a panel of chemicals across target proteins, in terms of binding strength and conformation to each chemical-protein pocket pair [47]. Both DRAR-CPI and DDI-CPI servers are employed for computational drug repositioning by the CPI server [48, 49]. The molecular structure of strychnobiflavone was submitted to the DRAR-CPI and DDI-CPI servers, and parameters were set to the default values. Results were considered satisfactory when the algorithms presented positive consensual data.

## 3. Results

### 3.1. Target Fishing Approach

**Figure 1:**
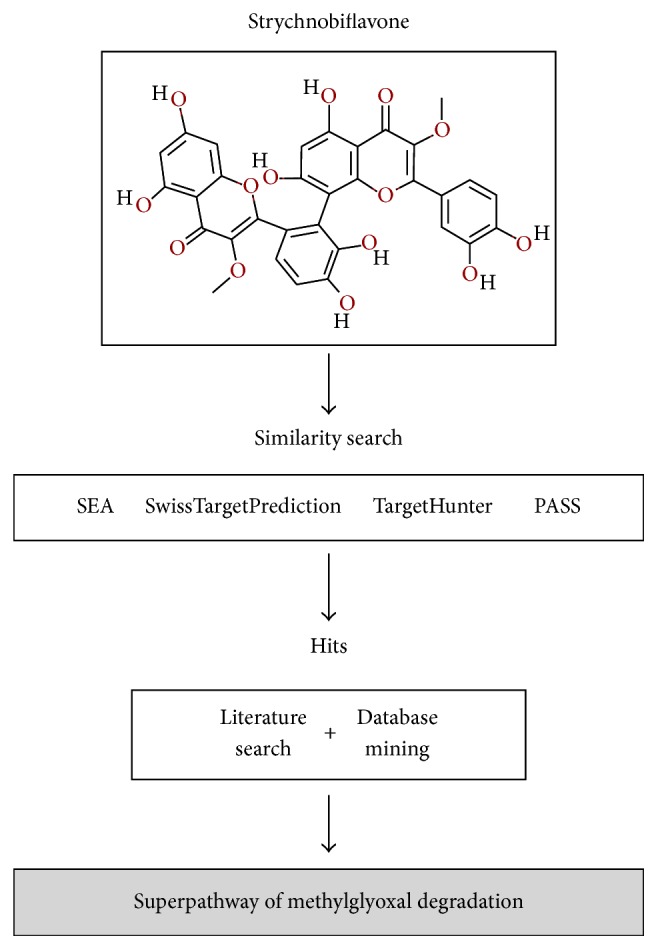
Computational framework used in the target fishing prediction of strychnobiflavone in the* Leishmania infantum* metabolic superpathway.

The molecular structure of strychnobiflavone was analyzed by distinct bioinformatics programs, aiming to screen the metabolic pathway of this molecule on* L. infantum*, as well as its molecular target in these parasites. For this, the structure of the flavonoid was evaluated by applying distinct algorithms, which used chemical similarity to identify proteins with known ligands to show similarity to this molecule [50]. In the results, the TargetHunter, SwissTargetPrediction, SEA, and PASS servers identified 21, 15, 75, and 630 putative targets, respectively. A positive consensual result was obtained with three hits: NADPH oxidase, Aldose reductase, and Aldo-keto reductase, which were employed as keywords for a literature review. The aim was to perform an evaluation of cross-reference between these terms and* Leishmania* proteins, as well as to search for references about their involvement in the parasite's biology. The following strategies were entered in the PubMed server: (“NADPH oxidase” [MeSH Terms] OR (“NADPH” [All Fields] AND “oxidase” [All Fields]) OR “NADPH oxidase” [All Fields]) AND (“leishmanial” [MeSH Terms] OR “leishmanial” [All Fields]) for [“NADPH oxidase”], resulting in 35 references founded; and (“aldehyde reductase” [MeSH Terms] OR (“aldehyde” [All Fields] AND “reductase” [All Fields]) OR “aldehyde reductase” [All Fields] OR (“aldose” [All Fields] AND “reductase” [All Fields]) OR “aldose reductase” [All Fields]) AND (“leishmanial” [MeSH Terms] OR “leishmanial” [All Fields]) for [“Aldose reductase”], resulting in eight identified references. In the case of [“Aldo-keto reductase”], only one reference was found. Data were extracted, analyzed, and compared with the metabolic pathway information present in the KEGG and MetaCyc servers. The results showed that the mechanism of action of strychnobiflavone was based on the inhibition of the methylglyoxal degradation superpathway (Figure 1).

### 3.2. STRING Analysis and Sequence Homology

The distribution of Glyoxalase system proteins in* L. infantum* showed the presence of Glyoxalase I (EC 4.4.1.5), Glyoxalase II (EC 3.1.2.6), and Aldo-keto reductase (EC 1.1.1.21) proteins [51]. The amino acid sequences of these antigens were submitted to a STRING analysis, and nine sequences were identified to interact with Glyoxalase I or Glyoxalase II proteins, whereas 10 sequences were identified to interact with Aldo-keto reductase. Since strychnobiflavone presents low toxicity in mammalian cells [19], one could speculate that its target is absent or expressed in low levels in these cells. Next, a homology analysis against human proteins was performed, and six sequences related to the Glyoxalase proteins were selected by their significant distinction with their homolog in mammalians (Table 1). These amino acid sequences were then selected for further analysis.

### 3.3. Molecular Modeling, Druggability, and Docking Assay

The structural prediction of a protein is performed by means of bioinformatics programs and theoretical chemistry, which is required, given that protein functions are dependent on their defined chemical structure [52]. In this sense, the six previously selected sequences were submitted to an automated homology model using the I-TASSER server, and, based on the* c*-scores, the best model was selected (Table 2). In addition, binding sites were detected in the screening models and were analyzed in terms of both their geometrical and their physicochemical properties. Ligands generally create favorable interactions with their binding sites; in this context, the active binding site of a hypothetical protein (UniProt ID: A4I8D6), which presented a drug score and a simple score of 0.81 and 0.62, respectively, showed the best results (Table 2). To confirm these findings, a docking analysis was performed by using the SwissDock server, in which the FullFitness and Gibbs free energy (Δ*G*) parameters were evaluated. The results showed that strychnobiflavone showed affinity with a highest druggability score and a FullFitness of −2985.25 kcal/mol, besides an estimated Δ*G* of −8.67 kcal/mol (Table 2). Since the sequence of this protein was annotated as a hypothetical protein, it was submitted to a functional annotation. In the results, this was identified as a hydrolase-like protein, with accuracy, sensitivity, and specificity values of 78.5%, 78.5%, and 100%, respectively.

### 3.4. Chemical-Protein Interactome Profile

The drug adverse reactions are undesirable, and since they can be caused by unexpected chemical-protein interactions, it is reasonable to predict interactions based on the mining of the chemical-protein interactome (CPI) [53]. In this sense, DRAR-CPI and DDI-CPI servers were used to screen undesired interactions between strychnobiflavone and human proteins (Table 3), as well as between strychnobiflavone and other drugs (Table 4). The results showed that this molecule can interact with an alcohol dehydrogenase class-3 protein, whereas no interaction was found between this molecule and evaluated drugs.

## 4. Discussion

Flavonoids represent an important family of polyphenolic compounds that exist in plants, vegetables, and fruits. Since people use substantial amounts of these molecules daily, it is accepted that flavonoids are not toxic to humans [54]. Recently, a flavonoid derived from* Strychnos pseudoquina* stem bark, namely, strychnobiflavone, presented an effective antileishmanial activity against* L. amazonensis* and* L. infantum* promastigotes and amastigotes. In addition, the mechanism of action of this molecule in* L. infantum* was evaluated and proved to be related to alterations in the parasite's mitochondrial membrane [19, 20]. In this context, the aim of the present study was to employ distinct bioinformatics programs to screen the metabolic pathway targeted by strychnobiflavone in* L. infantum* parasites.

The use of* Leishmania* promastigotes and amastigotes in* in vitro* studies to identify new antileishmanial products is still a key strategy in the development of new drugs [55]. However, it is not an easy task, since studies have shown the* in vitro *and/or* in vivo *biological action upon the parasites, but no mechanism of action has been proven. In this context, distinct bioinformatics strategies, such as target fishing and molecular docking assays, could be employed as technologies able to screen biological targets of distinct molecules in parasites, since they are based on the analysis of chemical structures by using information from biologically annotated databases, thus aiding many research groups [50].

Regarding the present study's results, changes in the parasite's metabolism were associated with three major enzymes related to the methylglyoxal degradation superpathway in* L. infantum*: Glyoxalase I, Glyoxalase II, and Aldo-keto reductase, which were evaluated by a STRING server. In this regard, the homology search performed among the selected sequences showed that six sequences presented significant differences between* Leishmania* and human proteins. Among them, a hypothetical protein (UniProt ID: A4I8D6), able to interact with Glyoxalase II, showed the highest druggability and molecular docking score and could be considered a possible molecular target for strychnobiflavone in* L. infantum*.

The methylglyoxal degradation superpathway has been also suggested to be a metabolic target of other chemotherapeutic agents against* Plasmodium falciparum*,* Toxoplasma gondii*,* L. major*,* Trypanosoma brucei*,* Trypanosoma cruzi*,* Entamoeba histolytica*, and* Giardia lamblia *[51, 56]. As a consequence, and due to the high similarity between* Leishmania *and* Trypanosoma* genus parasites, one could speculate that our computational approach was valid in identifying the possible biological target of strychnobiflavone in* L. infantum*.

*Leishmania* proteome information indicates that between 50% and 65% of all protein sequences have yet to be reported clearly [57] and are consequently classified as “uncharacterized or hypothetical” due to the fact that they present a low identity to known protein sequences [58]. The lack of identity with other sequenced organisms could be explained by the fact that, in the past,* Leishmania* was phylogenetically differentiated from the higher eukaryotes [59]. Thus, the hypothetical protein sequence identified here was submitted to an in silico functional annotation protocol, and results showed that it was predicted to be a hydroxylase-like protein. In this context, the data obtained in this study suggest the involvement of strychnobiflavone in the methylglyoxal degradation superpathway, due to its interaction with Glyoxalase II. In addition, the use of a CPI server, together with biology-based integrative systems, showed that no significant interaction with human proteins was found, then suggesting the absence of side effects if strychnobiflavone was used to treat human leishmaniasis.

In conclusion, it was proved strychnobiflavone interacts with the alcohol dehydrogenase class-3 protein, and results showed that the putative metabolic pathway inhibited by the molecule in the parasites was the methylglyoxal degradation superpathway, with a hydrolase-like protein proving to be the molecular target in* Leishmania*. Due to similar findings in other trypanosomatids, it could be speculated that our strategy, using distinct bioinformatics tools, was valid and could be well employed to screen other biological targets evoked by distinct molecules in different pathogens. In addition,* in vitro* biological studies are currently under development to confirm our findings, and preliminary results have shown that strychnobiflavone does act on the methylglyoxal degradation superpathway in* L. infantum*.

## Figures and Tables

**Table 1:** List of proteins related to the methylglyoxal degradation superpathway in *Leishmania infantum* and the sequence homology regarding mammalian proteins. N.C.: not calculated; N.F.: not found.

UniProt ID	Protein name	STRING score	BLASTp			
			*Homo sapiens*		*Mus musculus*	
			*E* value	Identity %	*E* value	Identity %
A4IBI9	Glyoxalase I	N.C.	3*E* − 16	35	6*E* − 16	34
A4I2L1	Putative methylmalonyl-coenzyme a mutase	0,974	0*E* + 00	62	0*E* + 00	62
A4I7A2	Putative acetyl-CoA carboxylase	0,946	0*E* + 00	37	0*E* + 00	36
A4I399	Putative propionyl-CoA carboxylase beta chain	0,867	6*E* − 80	68	4*E* − 77	68
A4I398	Putative propionyl-CoA carboxylase beta chain	0,867	2*E* − 34	54	7*E* − 33	54
A4HUX0	Putative 3-methylcrotonyl-CoA carboxylase beta subunit	0,867	0*E* + 00	59	0*E* + 00	61
E9AG50	Kinase-like protein	0,848	4*E* − 64	39	5*E* − 65	38
A4HZ66	Metallo-beta-lactamase family-like protein	0,653	5*E* − 69	41	7*E* − 69	41
A4I7C0	Methylcrotonyl-CoA carboxylase biotinylated subunit protein-like protein	0,642	0*E* + 00	44	8*E* − 177	44
A4HRC6	Putative carboxylase	0,642	0*E* + 00	52	0*E* + 00	52
Q2PYN0	Glyoxalase II	N.C.	1*E* − 39	36	3*E* − 42	37
A4I309	D-lactate dehydrogenase-like protein	0,949	1*E* − 79	36	1*E* − 72	35
A4I1U1	Methylmalonyl-CoA epimerase-like protein	0,892	2*E* − 50	61	1*E* − 50	60
A4HSD8	NUDIX hydrolase dihydroneopterin triphosphate pyrophosphohydrolase/hydrolase	0,660	N.F.	N.F.	2,9	39
A4HW95	Putative glutathione-S-transferase/glutaredoxin	0,659	4*E* − 24	27	3*E* − 25	26
A4I8D6	Uncharacterized protein	0,631	3*E* − 08	29	8*E* − 08	29
A4I330	Obg-like ATPase 1	0,631	5*E* − 149	54	3*E* − 148	54
A4I2Y9	Putative GTP binding protein	0,631	7*E* − 60	34	7*E* − 61	35
E9AHF3	Glutaredoxin-like protein	0,609	1*E* − 14	33	3*E* − 16	34
A4HYU2	Putative glutaredoxin	0,609	3*E* − 08	29	3*E* − 08	27
A4I342	Aldo-keto reductase-like protein	N.C.	1*E* − 60	36	5*E* − 60	37
A4HY37	Gamma-glutamylcysteine synthetase	0,623	8*E* − 104	47	8*E* − 104	47
A4I8R5	Putative d-xylulose reductase	0,611	3*E* − 79	41	1*E* − 80	42
E9AG23	D-3-phosphoglycerate dehydrogenase-like protein	0,549	8*E* − 51	34	2*E* − 51	34
A4IAM3	D-isomer specific 2-hydroxyacid dehydrogenase-like protein	0,549	3*E* − 19	32	1*E* − 19	32
A4I9V3	D-isomer specific 2-hydroxyacid dehydrogenase-protein	0,549	2*E* − 13	32	5*E* − 12	34
A4IDE7	Putative aldehyde dehydrogenase	0,505	2*E* − 137	46	2*E* − 144	46
A4IDU0	Aldehyde dehydrogenase	0,493	3*E* − 59	32	6*E* − 58	30
A4I5W9	Putative aldehyde dehydrogenase	0,493	7*E* − 61	32	2*E* − 57	32
A4I1F4	Aldehyde dehydrogenase, mitochondrial	0,493	4*E* − 175	52	2*E* − 174	52
A4HRT1	Putative delta-1-pyrroline-5-carboxylate dehydrogenase	0,493	7*E* − 180	50	4*E* − 176	49

**Table 2:** List of selected proteins and their evaluation as putative molecular targets of strychnobiflavone.

UniProt ID	Molecular Modelling		Druggability		Molecular docking	
	PDB hit	*C*-score	Drug score	Simple score	FullFitness (kcal/mol)	Estimated Δ*G* (kcal/mol)
A4IBI9	2c21A	1.27	0.58	0.20	−498.92	−8.03
A4HSD8	2kdvA	−0.65	0.75	0.28	−501.41	−8.37
A4I8D6	4r04A	−0.82	0.81	0.62	−2985.25	−8.67
E9AHF3	3h8qA	0.26	0.73	0.08	−244.21	−7.40
A4HYU2	3uiwA	0.26	0.63	0.24	−265.49	−7.80
A4I9V3	1ygyA	−0.31	0.79	0.27	−632.91	−8.41

**Table 3:** List of human proteins interacting with strychnobiflavone predicted by the DRAR-CPI and DDI-CPI servers.

DDI-CPI				DRAR-CPI			
PDB ID	Class	Putative target	Docking score	PDB ID	Putative target	Docking score	*Z*′-score
3GJW	PD	Poly [ADP-ribose] polymerase 1	−10,6	1MC5	Alcohol dehydrogenase class-3	−612,03	−419,62
2WIJ	PD	Cholinesterase	−10,5	2BH9	Glucose-6-phosphate 1-dehydrogenase	−580,99	−416,22
1QTN	PD	Caspase-8	−10,4	1ORE	Adenine phosphoribosyltransferase	−614,95	−376,09
1O86	PD	Angiotensin-converting enzyme	−10,3	2CG5	L-aminoadipate-semialdehyde dehydrogenase-phosphopantetheinyl transferase	−649,52	−376,07
1LD8	PD	Protein farnesyltransferase subunit beta	−10,2	1ZKK	Histone-lysine N-methyltransferase SETD8	−630,85	−371,27
1T5A	PD	Pyruvate kinase isozymes M1/M2	−10,1	1LN1	Phosphatidylcholine transfer protein	−639,55	−370,72
1M13	PD	Nuclear receptor subfamily 1 group I member 2	−10,0	1J8F	NAD-dependent deacetylase sirtuin-2	−542,85	−339,43
1GZ4	PD	NAD-dependent malic enzyme, mitochondrial	−9,7	1HAK	Annexin A5	−513,05	−309,57
2PGJ	PD	ADP-ribosyl cyclase 1	−9,7	1AXN	Annexin A3	−533,45	−302,98
2FZE	PK	Alcohol dehydrogenase class-3	−9,5	1YOW	Steroidogenic factor 1	−593,18	−300,48

**Table 4:** List of drugs interacting with strychnobiflavone predicted by the DRAR-CPI and DDI-CPI serves.

DDI-CPI		DRAR-CPI		
Library drug	Confidence	Library drug	Association score	*P* value
Betamethasone acetate	1,0	Dalfopristin 2	1,00	0,03
Buprenorphine hydrochloride	1,0	Cromoglicate 2	−1,00	0,13
Candesartan cilexetil	1,0	Cisapride 6	−0,89	0,06
Ceftriaxone 2	1,0	Didanosine 2	−0,88	0,03
Ciclesonide 2	1,0	Darunavir 4	−0,84	0,20
Clarithromycin 2	1,0	Droperidol 2	−0,84	0,03
Clarithromycin 3	1,0	Aliskiren	0,16	0,83
Dextromethorphan 3	1,0	Indinavir 2	0,81	0,26
Dihydroergotamine 2	1,0	Droperidol 3	−0,79	0,11
Dihydroergotamine 3	1,0	Folic acid 4	−0,78	0,23
